# Receptor-Mediated Endocytosis of VEGF-A in Rat Liver Sinusoidal Endothelial Cells

**DOI:** 10.1155/2019/5496197

**Published:** 2019-09-10

**Authors:** Seyed Ali Mousavi, Frode Skjeldal, Marita Sporstøl Fønhus, Linda Hofstad Haugen, Winnie Eskild, Trond Berg, Oddmund Bakke

**Affiliations:** ^1^Department of Immunology and Transfusion Medicine, Akershus University Hospital, University of Oslo, Norway; ^2^Department of Biosciences, University of Oslo, Norway

## Abstract

**Background and Aims:**

Vascular endothelial growth factor (VEGF) receptors (VEGFR1 and VEGFR2) bind VEGF-A with high affinity. This study sought to determine the relative contributions of these two receptors to receptor-mediated endocytosis of VEGF-A and to clarify their endocytic itineraries in rat liver sinusoidal endothelial cells (LSECs).

**Methods:**

Isolated LSECs and radiolabeled VEGF-A were used to examine surface binding and receptor-mediated endocytosis. Quantitative real time RT-PCR (Q-RT-PCR) and Western blotting were applied to demonstrate receptor expression.

**Results:**

Q-RT-PCR analysis showed that VEGFR1 and VEGFR2 mRNA were expressed in LSECs. Ligand saturation analysis at 4°C indicated two different classes of [^125^I]-VEGFA binding sites on LSECs with apparent dissociation constants of 8 and 210 pM. At 37°C, LSECs efficiently took up and degraded [^125^I]-VEGF-A for at least 2 hours. Uptake of [^125^I]-VEGF-A by LSECs was blocked by dynasore that inhibits dynamin-dependent internalization, whereas inhibition of cysteine proteases by leupeptin inhibited degradation without affecting the uptake of [^125^I]-VEGF-A, suggesting that it is degraded following transport to lysosomes. Incubation of LSECs in the continued presence of a saturating concentration of unlabeled VEGF-A at 37°C was associated with a loss of as much as 75% of the total VEGFR2 within 30 min as shown by Western blot analysis, whereas there was no appreciable decrease in protein levels for VEGFR1 after 120 min incubation, suggesting that VEGF-A stimulation downregulates VEGFR2, but not VEGFR1, in LSECs. This possibility was supported by the observation that a hexapeptide that specifically blocks VEGF-A binding to VEGFR1 caused a marked reduction in the uptake of [^125^I]-VEGF-A, whereas a control peptide had no effect. Finally, live cell imaging studies using a fluorescently labeled anti-VEGFR2 antibody showed that VEGFR2 was transported via early and late endosomes to reach endolysosomes where degradation of the VEGFR2 takes place.

**Conclusion:**

Our studies suggest that, subsequent to VEGF-A binding and internalization, the unoccupied VEGFR1 may recycle to the cell surface allowing its reutilization, whereas the majority of the internalized VEGFR2 is targeted for degradation.

## 1. Introduction

Vascular endothelial growth factor A (VEGF-A) belongs to a family that in mammalian species comprises four other members denoted as VEGF-B, C, D and placenta growth factor (PlGF), each encoded by different genes. VEGF mRNA is expressed in most organs in the body including the liver [1]. VEGF-A pre-mRNA is alternatively spliced to yield at least seven related proangiogenic polypeptides, containing 121, 145, 148, 165, 183, 189, or 206 amino acid residues, which differ in terms of their bioavailability and their ability to regulate angiogenesis [2–4]. VEGF-A_165_ (hereafter referred to as VEGF-A) is a secreted homodimeric glycoprotein of ~38 kDa that binds with high affinity to two receptor tyrosine kinases, VEGFR1 (Flt-1) and VEGFR2 (KDR), which are predominantly expressed in blood vascular endothelial cells (ECs) including liver sinusoidal endothelial cells (LSECs) that line the hepatic sinusoids [5, 6]. Interaction of VEGF-A with cell surface VEGFR2 promotes receptor dimerization and trans-phosphorylation on multiple tyrosine residues that allows it to activate cytoplasmic signaling proteins. These in turn lead to a cascade of intracellular signaling pathways including phospholipase C-*γ*1 (PLC*γ*1), phosphatidylinositol 3-kinase (PI3-K), and mitogen-activated protein kinase (MAPK, ERK1/2) [7–9]. VEGFR2 is the primary receptor that in LSECs (as in other ECs) mediates the biological effects of VEGFA that is crucial to angiogenesis, including EC migration, proliferation, and tube formation. Angiogenesis may be initiated in several situations in the liver: In liver regeneration after various kinds of injury, after hepatectomy, and in liver metastases [10–14]. In normal liver fenestra is one of the distinctive characteristics of LSECs that allow circulating macromolecules, including lipoprotein particles and chylomicron remnants, to enter or leave the space of Disse [15]. Data also suggest that VEGF released by hepatocytes and hepatic stellate cells acting through VEGF receptors plays an important role in the maintenance of liver fenestrae in a nitric oxide-dependent manner [16].

Signaling through VEGFR1 apparently has no direct role in VEGF-A-induced angiogenesis in ECs since mice lacking the kinase domain of VEGFR1 develop normally [17]. However, VEGFR1 is essential for embryonic angiogenesis since VEGFR1 null mice die during embryonic development due to vascular overgrowth [18]. However, VEGFR1 (and its soluble form) binds VEGF-A with 10-fold higher affinity than VEGFR2, and by decreasing VEGF-A bioavailability at VEGFR2, VEGFR1 may serve as a decoy receptor [17, 19]. Nevertheless, at least in one context, regeneration of the liver following toxin-induced injury in mice, a role for VEGFR1 in mediating biological actions of VEGF-A has been identified. This involves production of hepatotrophic factors such as HGF (hepatocyte growth factor) by LSECs in response to VEGF-A released by hepatocytes, which in turn stimulate proliferation of hepatocytes [20, 21].

Although much is known about VEGFR2-mediated endocytosis of VEGF-A in cultured human umbilical vein endothelial cells (HUVECs) and bovine aortic endothelial cells (BAECs) [22, 23], little is known about the role of VEGFR1 in this process. LSECs perform many critical functions including removal of waste and potentially injurious molecules via endocytic processes mediated by receptors such as mannose receptor, scavenger receptors, and Fc*γ*RIIB2 [24, 25]. As yet, however, receptor-mediated endocytosis of VEGF-A has not been examined in these cells. In this study, we investigated receptor-mediated endocytosis of VEGF-A in isolated rat LSECs with a particular objective of determining the relative contributions of VEGFR1 and VEGFR2 to this process and the fates of ligand-bound VEGFR1 and VEGFR2 after internalization. To further clarify the traffic of VEGFR2 live cell imaging with fluorescently labeled monoclonal antibody was performed.

## 2. Material and Methods

### 2.1. Materials

The 165 amino acid form of VEGFA labeled with iodine-125 ([^125^I]-VEGF-A) (human recombinant, product number NEX 328) was obtained from PerkinElmer, Norway. Recombinant Rat VEGF (564-RV/CF) was obtained from R&D Systems. The VEGFR1-binding peptide (GNQWFI) and the control peptide (IFWQNG) were purchased from GenScript Inc., USA (SA1208). All other chemicals were from Sigma-Aldrich, unless otherwise indicated.

### 2.2. Preparation of LSECs from Rat Liver

Liver cells were isolated from male Wistar rats by the collagenase perfusion method [26]. LSECs were prepared by low speed differential centrifugation, and Percoll (Amersham Biosciences) density gradient centrifugation, followed by removal of Kupffer cells as described previously [24]. A yield of 40-50 × 10^6^ cells per liver was obtained. All procedures were conducted in accordance with institutional guidelines for maintenance and use of research animals.

### 2.3. Cell Culture Conditions

In preliminary experiments LSECs were seeded on fibronectin-coated or uncoated (plastic) 6-well plates. Control fibronectin-coated wells, consisting of [^125^I]-VEGF-A added to culture media in the absence of cells for 1 h, showed that more than 60% of the added radioactivity was associated with the wells, most likely due to the presence of VEGF-binding sites on fibronectin [27], making it difficult to obtain reliable estimates of the binding parameters. With cells plated in uncoated wells, although the background was considerably lower than with cells in fibronectin-coated wells, [^125^I]-VEGF-A binding could not be quantified accurately due to relatively poor adherence of LSECs to plastic culture plates as well as problems with cell detachment of cells during washing. Nonspecific binding was lowest when freshly isolated LSECs in suspension were used. For this reason and because background binding (≤10%) could be prevented, all binding and uptake experiments were performed with LSECs suspended in a balanced salt solution consisting of 145 mM NaCl, 5.4 mM KCl, 0.33 mM Na_2_HPO_4_, 0.34 mM KH_2_PO_4_, 0.8 mM MgSO_4_, 2 mM CaCl_2_, 20 mM HEPES [4-(2-hydroxyethyl)-1-piperazineethanesulfonic acid], pH 7.5, and 1% BSA (hereafter called the incubation buffer).

### 2.4. Cell Surface [^125^I]-VEGF-A Binding Assay

Suspensions of LSECs (4 × 10^6^ cells/ml) in incubation buffer were temperature-equilibrated in glass tubes for 30 min at 4°C and then incubated with [^125^I]-VEGF-A in concentrations ranging from 0.5 to 900 pM for 90 min with constant shaking. The binding was terminated by transferring 0.5 ml aliquot to centrifuge tubes which previously were filled with 4.5 ml of cold phosphate-buffered saline (PBS) containing 1% BSA and centrifuged. After washing the cells twice more, cell pellets were transferred to counting tubes and the surface-associated radioactivity was determined using a *γ*-counter. A 200 *μ*l aliquot of the cell suspension was also saved for calculating the radioactivity actually added to each tube. All binding values are corrected for nonspecific binding measured in the presence of a 100-fold excess unlabeled VEGF-A. Corrections were also made for the total radioactivity in the incubation tube.

### 2.5. Endocytosis and Degradation of [^125^I]-VEGFA

Suspensions of LSECs (4 × 10^6^ cells/ml) in incubation buffer were temperature-equilibrated in glass flasks for 15 min in a water bath at 37°C and then incubated with [^125^I]-VEGF-A to a final concentration of ~40 pM with constant shaking. At each time point, 2 × 200 *μ*l aliquot was withdrawn and transferred to centrifuge tubes which previously were filled with 4.8 ml of cold PBS/1% BSA and centrifuged. After centrifugation, 2 × 1 ml samples from the supernatants were saved and the remainder was aspirated. After washing twice more by resuspension in PBS/BSA, the cells were solubilized in 1.0 ml of 0.2 N NaOH and then transferred to counting tubes. Cell lysates and supernatants from the first wash were precipitated with trichloroacetic acid (TCA) and PBS/BSA at final concentrations of 10% and 0.25%, respectively, and then incubated on ice for 15 min. TCA-precipitable radioactivity (intact and/or partially degraded VEGF-A) was separated from TCA-soluble radioactivity (degradation products) by centrifugation at 4°C and the radioactivity associated with each fraction was determined using a *γ*-counter. Total cellular uptake of [^125^I]-VEGFA is expressed as the sum of cell-associated (TCA-precipitable plus TCA-soluble) radioactivity and TCA-soluble radioactivity released into the medium. For each experiment, a zero time (t = 0 min) value was determined immediately after the addition of [^125^I]-VEGF-A to the cell suspension and immediately washing the cells as above. The average values of surface-associated radioactivity at the zero time were subtracted from total cell-associated radioactivity to determine LSEC specific internalization of [^125^I]-VEGFA. The average values of TCA-soluble radioactivity at the zero time were subtracted from the total TCA-soluble radioactivity in the medium to quantify LSEC specific degradation of [^125^I]-VEGFA. The radioactivity of the zero time samples was typically ≥97% TCA-precipitable. A 200 *μ*l aliquot of the cell suspension was also saved to correct for the actual amount of radioactivity present in each flask. For inhibitor studies, cells were treated with dynasore (40 *μ*M), concanamycin A (2 *μ*M), or leupeptin (2 mM) at 37°C for 5-10 min and subsequently incubated with [^125^I]-VEGFA for 90 min at 37°C in the continued presence of inhibitor. Control cells were similarly incubated with [^125^I]-VEGFA in the presence of vehicle only [DMSO (≤1%) or PBS]. Cells were then washed and TCA-soluble and TCA-precipitable radioactivity were determined as described above.

### 2.6. Western Blot Analysis

Suspensions of LSECs (6 × 10^6^ cells/ml) were incubated for different times with unlabeled VEGF-A (40 *μ*g/ml = 1 nM) in the presence of cycloheximide (20 *μ*g/ml). Cells were washed with cold PBS (without BSA) and then transferred to a micro-centrifuge tube. Whole-cell lysates (~20 *μ*g) were prepared in RIPA buffer [50 mM Tris-HCl, pH 7.5, 1% Nonidet P-40, 0.25% sodium deoxycholate, 0.05% sodium dodecyl sulfate (SDS), 150 mM NaCl, 1 mM EDTA, supplemented with protease inhibitors]. Proteins were separated using SDS-PAGE (4-20% gels, BioRad), transferred to a polyvinylidene fluoride (PVDF) membrane (IPVH00010, Millipore) and incubated overnight at 4°C with goat anti-VEGFR2 antibody (sc-393179, Santa Cruz Biotechnology, 1:100). The membrane was washed three times in 0.1% Tween20/PBS and then incubated with mouse IgG linked horseradish peroxidase- (HRP-) conjugated secondary antibody (NA931, GE Healthcare, 1:5000) for 60 min. Protein bands were detected using the ECL plus Western blotting detection system (RPN2232, GE Healthcare). The blot was stripped (Stripping buffer, 21059, Thermo Fisher Scientific) and reprobed with rabbit anti-VEGFR1 antibody (ab32152, Abcam, 1:1000), then with mouse anti-*α*-tubulin (T9026, Sigma-Aldrich, 1:10000), which served as loading control for total cell lysates. Proteins bands were detected as described above.

### 2.7. Real Time Quantitative Reverse Transcription Polymerase Chain Reaction (RT-PCR)

Total RNA was isolated from the cells using PerfectPure RNA Cultured Cell Kit (5Prime). The cDNA was prepared by reverse transcription reaction using Superscript II RNase H^−^ Reverse Transcriptase (Invitrogen) and oligo dT_(15)_ primers according to the manufacturer's protocols (DNA Technology A/S). All primers used were designed by using the Primer3 Output program (http://bioinfo.ut.ee/primer3/). *β*-actin mRNA level was used as internal control for normalization. Primers for VEGFR1 were FWD, 5′-tcaccacggacctcaataca-3′; REV, 5′-cgatgcttcacgctgataaa-3′; for VEGFR2: FWD, 5′-ggaaggttgcttgctctcac-3′; REV, 5′-cagggcagacaagtgggtat-3′; and for *β*-actin: FWD, 5′-agccatgtacgtagccatcc-3′; REV, 5′-ctctcagctgtggtggtgaa-3′.

### 2.8. Live Cell Imaging

LSECs were seeded in 35-mm glass-bottom dishes (MatTek Corporation) at a density of 300 000/dish in serum-free DMEM medium (Invitrogen) at 37°C and 5% CO_2_ for 3 hours. Cells (~80% confluent) were washed twice with PBS and temperature-equilibrated in fresh medium for 20 min at 4°C and were then incubated at 4°C for 60 min with a monoclonal antibody raised against the extracellular domain of VEGFR2 (sc-57135, Santa Cruz Biotechnology), labeled with Alexa Flour 488 (Invitrogen). After washing to remove unbound Ab, the cells were incubated in fresh medium in the presence of unlabeled VEGF-A and LysoTracker red (Invitrogen) for an additional 0-360 min at 37°C. Cells were imaged by spinning disk confocal microscopy (Andor Technology).

### 2.9. Data Analysis

Statistical significance was assessed using unpaired t-test and one-way analysis of variance (ANOVA) followed by Dunnett's post hoc multiple comparison test. Differences were considered significant if p < 0.05. Statistical analyses were performed using Prism 5.0.1 (GraphPad Software Inc., CA). Unless otherwise specified, results shown are the mean ± standard deviation (SD) of three separate cell preparations, each with duplicate measurements.

## 3. Results

### 3.1. Quiescent Rat LSECs Express VEGFR1 and VEGFR2

**Figure 1:**
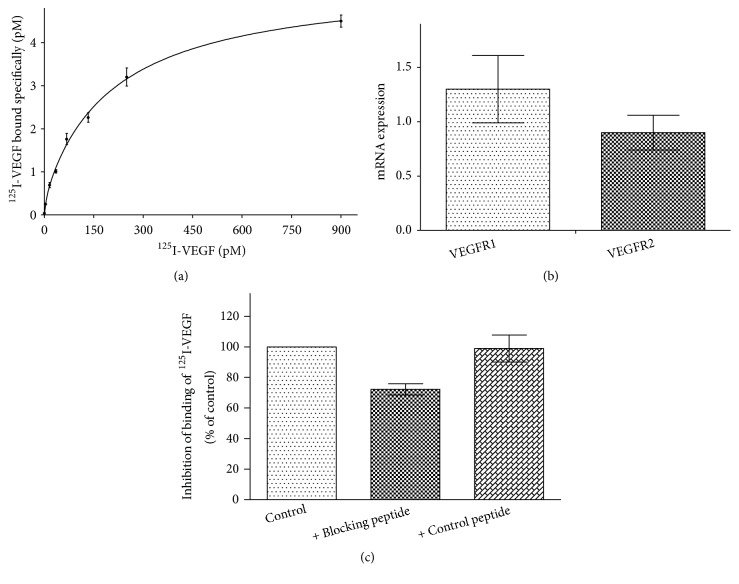
*Expression of VEGF receptors in LSECs. *(a)* Saturation binding of [*^125^*I]-VEGF-A to LSECs*. LSECs (4 × 10^6^/ml) were incubated with increasing concentrations of [^125^I]-VEGF-A (0.5-900 pM). After 90 min at 4°C, specific binding was determined as described in “Materials and Methods.” The curve was fitted using nonlinear regression with GraphPad Prism. Data represent the mean ± range of duplicate measurements from a representative experiment performed three times with similar results. (b)* Quantification of VEGFR1 and VEGFR2 mRNA expression*. After total RNA extraction and cDNA amplification, mRNA levels were quantified by real time RT-PCR as described in “Materials and Methods.” *β* actin mRNA level was used as internal control for normalization. Data represent the mean ± SD from three independent experiments. No statistical difference was found between the expression levels of VEGFR1 and VEGFR2 using unpaired t-test (p = 0.12). (c)* Effect of the VEGFR1-binding peptide on cell surface binding of [*^125^*I]-VEGF-A*. LSECs (4 × 10^6^/ml) were preincubated at 4°C with the VEGFR1-binding peptide (400 *μ*M in DMSO) or an equal concentration of the control peptide and then incubated with [^125^I]-VEGF-A. After 90 min at 4°C, specific binding was determined as described in “Materials and Methods.” Data represent the mean ± range from two independent experiments.

Initial experiments designed to evaluate binding activity showed that cell surface binding of [^125^I]-VEGF-A (~40 ng/ml = 1 nM) reached equilibrium after 90 min at 4°C and remained unchanged for at least 60 min (data not shown). Binding of [^125^I]-VEGF-A to LSECs at 4°C was specific since total binding could be reduced by ~80-90% in the presence of 100-fold molar excess of unlabeled VEGF-A (data not shown). The saturability of binding sites was shown by incubating LSECs for 90 min at 4°C with increasing concentrations of [^125^I]-VEGF-A (Figure 1(a)). Scatchard plots used to estimate the apparent dissociation constant (K_d_) and the number of surface binding sites per cell (B_max_) was biphasic (data not shown), suggesting the presence of two binding sites for VEGF-A on LSECs: a high affinity, low capacity receptor exhibiting an average K_d_ of 8±4 pM and B_max_ of 48±14 sites per cell, and a lower affinity, higher capacity receptor exhibiting an average K_d_ of 210±60 pM and B_max_ of 410±75 sites per cell (n = 3). These affinities are in the same range as those reported for VEGFR1 and VEGFR2 (10-26 pM versus 75-770 pM) in a variety of cell types [5, 28, 29]. However, rat LSECs seem to express less VEGF receptors on their surface compared with other endothelial cell types [29, 30].

Real time quantitative RT-PCR demonstrated the presence of VEGFR1 and VEGFR2 mRNAs in quiescent LSECs (Figure 1(b)). Consistent with previous results showing that VEGFR1 mRNA in rat LSECs is expressed more highly than VEGFR2 [31], the expression of VEGFR1 mRNA was about 1.6-fold higher than that of VEGFR2, but this difference was not statistically significant (p = 0.12). Western blot analysis on whole-cell lysates confirmed the expression of VEGFR1 and VEGFR2 at the protein level (lines 1 in Figure 3(a)).

It has been shown that a hexapeptide (GNQWFI) that binds VEGFR1 can inhibit VEGF-A binding to HUVECs [32]. We examined whether this peptide is able to block binding of [^125^I]-VEGF-A on the surface of LSECs. When LSECs were preincubated for 15 min at 4°C with either the blocking peptide or the no-blocking control peptide (IFWQNG) and then incubated with a saturating concentration of [^125^I]-VEGF-A (1 nM) for 90 min at 4°C, the radioactivity bound to cells treated with the blocking peptide was decreased by 20% compared with only 3% in cells treated with the control peptide (Figure 1(c)). These results confirm VEGFR1 involvement in the binding of [^125^I]-VEGF-A to LSECs.

### 3.2. Uptake and Degradation of [^125^I]-VEGF-A by LSECs Occurs via Receptor-Mediated Endocytosis

**Figure 2:**
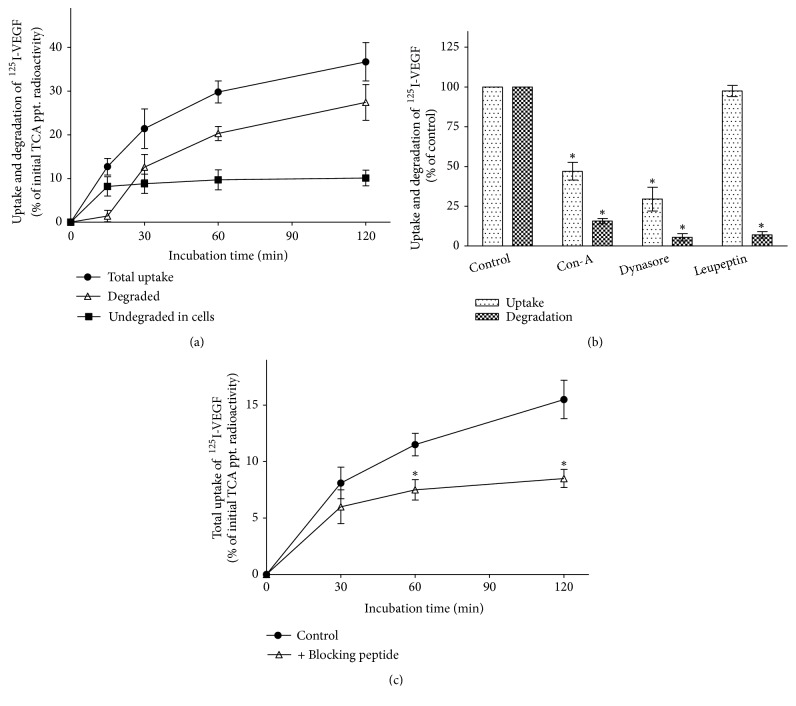
*Uptake and degradation of [* ^125^ *I]-VEGF-A by LSECs*. (a)* Time course of [*^125^*I]-VEGF-A uptake and degradation*: LSECs (4 × 10^6^/ml) were incubated at 37°C in medium containing [^125^I]-VEGF-A (40 pM). At the indicated times, duplicate aliquots were removed, and cells and medium were assayed for TCA-soluble and TCA-precipitable radioactivity as described in “Materials and Methods.” Degradation was calculated as the sum of the cell-associated and released TCA-soluble radioactivity at each time point. The specific uptake (per 4 × 10^6^ cells) at each time point was determined by adding up the released TCA-soluble radioactivity to the total cell-associated radioactivity and then subtracting the cell-associated radioactivity at the zero-time and expressed as a percentage of total TCA-precipitable (TCA-ptt.) radioactivity initially added to the cell suspension. Data represent the mean ± SD (n = 3). (b)* Effects of the endocytic pathway inhibitors on uptake and degradation of [*^125^*I]-VEGF-A*. LSECs (4 × 10^6^/ml) were pretreated with inhibitors or vehicle controls [DMSO for dynasore and concanamycin A (Con-A), PBS for leupeptin] at 37°C for 10 min prior to the addition of [^125^I]-VEGF-A (40 pM). The cells were then further incubated at 37°C for 90 min in the continued presence of inhibitor or vehicle. The specific uptake was determined as described for (a). Data are expressed as the percentage inhibition relative to control (vehicle-treated) cells. Concentrations of inhibitors employed were as follows: dynasore (40 *μ*M), Con-A (0.1 *μ*M), and leupeptin (50 *μ*g/ml). Data represent the mean ± SD (n = 3). Asterisk (*∗*) denotes statistically different from control (p < 0.001 by one-way ANOVA and Dunnett's post hoc test among control and treated groups with various inhibitors). (c)* Effect of the VEGFR1-binding peptide on [*^125^*I]-VEGF-A uptake*. A preincubation step of 5 min at 37°C was performed in the presence or absence of the VEGFR1-binding peptide (400 *μ*M in DMSO) prior to addition of [^125^I]-VEGF-A (1 nM). The cells (4 × 10^6^/ml) were then further incubated at 37°C for the indicated times in the continued presence or absence of the peptide. The specific uptake (per 4 ×10^6^ cells) at each time point was determined as described for (a). For the sake of simplicity, only the specific uptake is shown. Data represent the mean ± SD (n = 3). *∗*p < 0.03 vs. control by unpaired t-test. No statistical difference was found between conditions at 30 min using unpaired t-test.

The capacity of rat LSECs for specific uptake and degradation of VEGF-A was assessed by incubating of the cells in the continuous presence of [^125^I]-VEGF-A (~40 pM) over 120 min at 37°C. As shown in Figure 2(a), TCA-soluble radioactivity began to appear in the media after a lag phase of about 15 min and increased with continued incubation, whereas cell-associated radioactivity plateaued by 15 min. Most (≥90%) of the cell-associated radioactivity was TCA-insoluble at all time points, indicative of an intracellular location. The release of TCA-soluble radioactivity into the medium was temperature dependent because no degradation of [^125^I]-VEGF-A was observed at 4°C regardless of incubation time periods (data not shown). By 120 min about 35% of added [^125^I]-VEGF-A had been taken up by cells, while as much as 85-90% of the internalized ligand was degraded and released into medium as TCA-soluble radioactivity.

These results are consistent with a model in which the binding of [^125^I]-VEGF-A to its receptors on the LSEC is followed by the internalization of ligand-receptor complexes and subsequent degradation of the internalized ligand in endolysosomes. To examine the endocytic transport steps of VEGF-A uptake in LSECs, we tested the effects of three known inhibitors on receptor-mediated internalization and degradation of VEGF-A (Figure 2(b)). Dynasore, a selective and cell-permeable inhibitor of dynamin GTPase [33], has been shown to inhibit the internalization of VEGFR2 in HUVECs resulting in its accumulation on the plasma membrane [34]. We also found that this compound produced more than 70% inhibition of [^125^I]-VEGF-A uptake in LSECs at 40 *μ*M (Figure 2(b)) as compared with vehicle-treated control cells (p <0.001), indicating a dynamin-dependent entry route. Concanamycin A (Con-A) is a selective blocker of endosome/lysosome acidification by inhibiting the vacuolar-type H^+^-ATPase activity [35], whereas leupeptin is an inhibitor of lysosomal cysteine proteinases. In the presence of Con-A (0.1 *μ*M), both uptake and degradation were markedly reduced (by 50% and 80%, respectively) as compared with vehicle-treated control cells (p <0.001 for both comparisons). These results indicate that the internalized [^125^I]-VEGF-A remains associated with its receptor in neutralized early/sorting endosomes, and the occupied receptor is not available for binding of new ligand. In contrast, leupeptin (50 *μ*g/ml) profoundly inhibited [^125^I]-VEGFA degradation (p <0.001) without affecting the total uptake, thus causing an intracellular accumulation of undegraded ligand. These results confirm that the production of TCA-soluble radioactivity in the media was due to specific protease activity within the lysosomes, rather than unspecific extracellular degradation of [^125^I]-VEGFA.

### 3.3. Analysis of the Relative Role of VEGF Receptors in Mediating VEGF-A Endocytosis

The results presented in Figure 2(a) show that after 2 h incubation at 37°C, LSECs were able to internalize ~5 times more ligand than what can bind to the cell surface at 4°C (~2000 ligand molecules were estimated to be taken up by each cell at 37°C, compared to ~450 ligand molecules that could bind at 4°C). This observation is consistent with either repeated recycling of receptors or a redistribution of receptors from internal pools to the cell surface or insertion of newly synthesized receptors into the plasma membrane. Internalization of ligands (125I-VEGFA) is mediated by both VEGFR1 and VEGFR2 and to explain the extent of recycling it is of interest to determine the capacities of the two receptors to mediate uptake and to what extent these receptors are recycled or downregulated following ligand binding.

To determine the contribution by VEGFR1 to the uptake of VEGFA in the LSECs, cells were incubated at 37°C with either 1 nM [^125^I]-VEGF-A alone, or with [^125^I]-VEGF-A plus the VEGFR1-binding peptide. As shown in Figure 2(c), after 30 min incubation in the presence of the VEGFR1-binding peptide, about 6% of added [^125^I]-VEGF-A had been taken up and degraded by cells and by 120 min 8,5% of added ligand had been taken up and degraded by cells. In contrast, the amount of [^125^I]-VEGF-A taken up by control cells (i.e., in the absence of the VEGFR1-binding peptide) increased with continued incubation, and by 120 min the amount of ligand specifically taken up by cells was approximately 2 times greater than in treated cells (15.5% vs. 8.5% of added ligand). No decrease in uptake was seen in cells that were coincubated with the control peptide (data not shown), suggesting a selective blockade of VEGF-A endocytosis via VEGFR1. Treatment of cells with cycloheximide (20 *μ*g/ml) caused a further slight decrease in the uptake of [^125^I]-VEGF-A: ~13% of the added ligand was taken up after 120 min incubation at 37°C compared to ~15.5% in control cells (data not shown). These results suggest that the gradual decrease in uptake of [^125^I]-VEGF-A in control cells is due to downregulation of VEGFR2 at the cell surface, whereas VEGFR1 may recycle to the plasma membrane and thereby continue to participate in VEGF-A endocytosis.

### 3.4. VEGF-A Stimulation Downregulates VEGFR2, but Not VEGFR1, in LSECs

**Figure 3:**
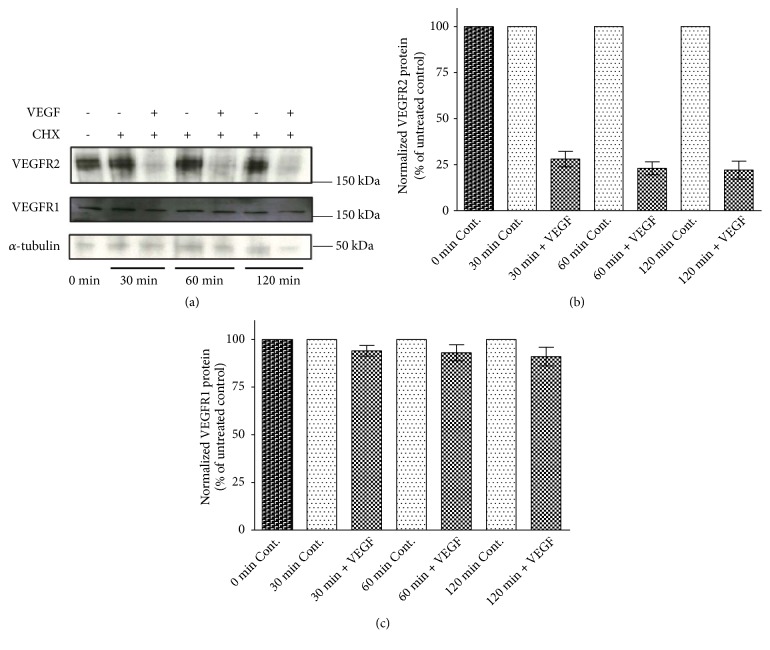
*Effect of 1 nM unlabeled VEGF-A on protein levels of VEGFR1 and VEGFR2*. LSECs (4 × 10^6^/ml) were treated with or without unlabeled VEGF-A (1 nM) in the presence of cycloheximide (CHX, 20 *μ*g/ml) at 37°C for the times indicated and protein levels were analyzed by immunoblotting as described in “Materials and Methods.” (a) Shown are representative blots of two independent experiments. Molecular weight markers are indicated on the side. VEGFR2 ran as a band of approximately 230 kDa. VEGFR1 was present at approximately 180 kDa. ((b) and (c)) Pooled data from two experiments were quantified by densitometry for VEGFR1 and VEGFR2 blots and expressed as percent of untreated control after correction with *α*-tubulin. Data represent the mean ± range from two experiments.

Previous studies have shown that VEGFR2 is a target for ligand-induced degradation [34, 36]. To determine if VEGF receptors in LSECs would be degraded following treatment with VEGF-A, immunoblot analysis of the cell lysates from LSECs that were treated with or without 1 nM unlabeled VEGF-A for 120 min at 37°C was performed (Figure 3). After 30 min incubation at 37°C, VEGFR2 levels were reduced significantly by VEGF-A to 28±4.2% of control, but little decrease occurred after this time (Figures 3(a) and 3(b)). In contrast, VEGFR1 levels after 120 min exposure to VEGF-A were reduced to 91±4.9% of control (Figures 3(a) and 3(c)). The results presented in Figures 3(a)–3(c) suggest that the time course and extent of degradation of VEGFR2 and VEGFR1 in LSECs could account for the differences in [^125^I]-VEGF-A uptake in the presence and absence VEGFR1-binding peptide (observed in Figure 2(c)), providing further evidence that the majority of internalized VEGFR1, but not VEGFR2, can recycle back to the plasma membrane and then be available for further uptake. However, given the very low number of cell surface VEGFR1 (48±14 sites/cell) the receptors must be recycled and additional VEGFR1 from preexisting intracellular pools must be recruited to the plasma membrane to account for the continued uptake of [^125^I]-VEGF-A under the saturating concentrations of the ligand in control cells.

### 3.5. Internalization and Intracellular Trafficking of Surface-Bound Anti-VEGFR2 Ab

**Figure 4:**
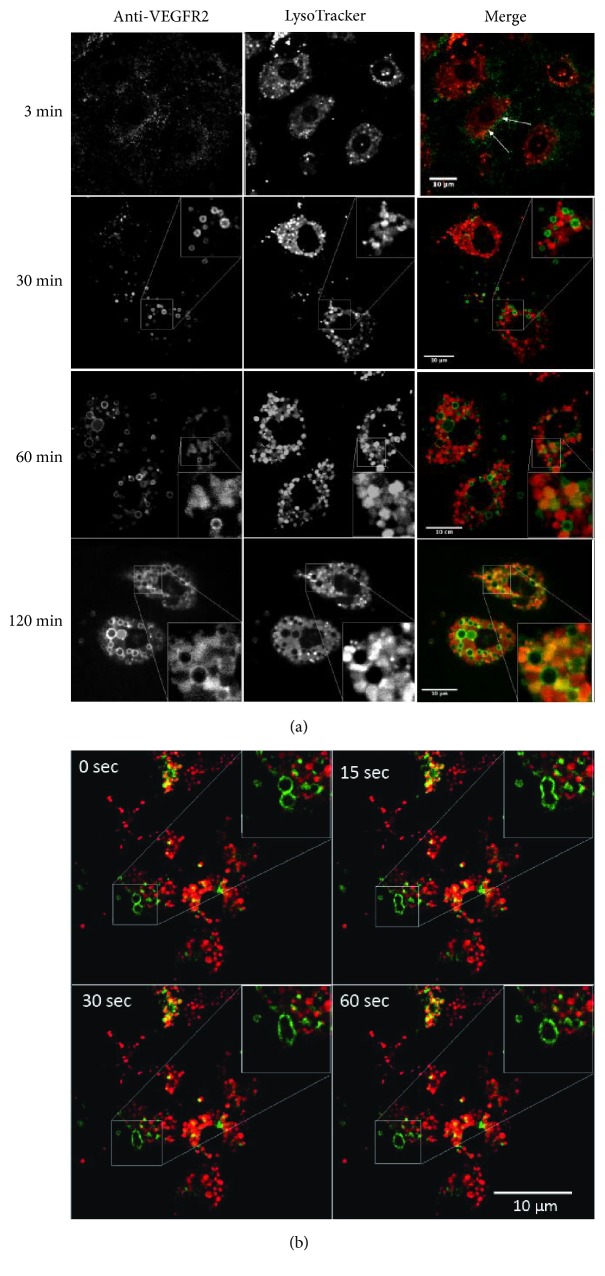
*Monitoring of intracellular trafficking of anti-VEGFR2 Ab by confocal microscopy*. After allowing fluorescently labeled anti-VEGFR2 Ab to bind to adherent LSECS (~80% confluent) for 60 min at 4°C, unbound Ab was washed away, and the cells were allowed to internalize surface-bound anti-VEGFR2 at 37°C in the presence of unlabeled VEGF-A and LysoTracker Red (LTR). (a) Shown are representative fluorescence images from each time point (n = 3). Images in each column in the top panel show anti-VEGFR2 antibody (left), LTR (center), and fluorescence overlay (right). In the merge images green color = anti-VEGFR2 antibody, red = LTR, and yellow = their colocalization, highlighting the gradual accumulation of internalized anti-VEGFR2 within acidic compartments (late endosomes/lysosomes). (b) The bottom panel shows live cell imaging of a fusion event.

To further clarify the trafficking of VEGFR2 in LSEC live cell imaging was performed to investigate the intracellular trafficking of a fluorescently labeled monoclonal antibody at 37°C following its binding to VEGFR2 at 4°C. To determine whether VEGFR2 reached acid organelles we added LysoTracker Red (LTR) (a marker for acidic endocytic compartments) to the cells. As shown in Figure 4(a), anti-VEGFR2 was mainly localized at the cell membrane at early time points (3 min). After 30 min, anti-VEGFR2 was localized in small endocytic vesicles that were scattered throughout the cytoplasm. These vesicles were of different size, and the receptor was localized along the limiting membrane of the vesicles suggesting neutral pH. LTR localized to intracellular vesicles, but the majority of the internalized VEGFR2, was detected in LTR negative vesicles, probably early endosomes. After 60 min anti-VEGFR2 appeared in a population of endocytic structures that were even larger in size dispersed throughout the cytoplasm, and we could also detect a small but increasing amount of anti-VEGFR2 colocalizing with LTR, indicative of more intraluminal sorting for degradation. However, large amount of anti-VEGFR2 was still localized to the limiting membrane of the vesicles. The large endosomes seemed to be ring-shaped, suggesting that the labelled antibody was attached to VEGFR2 on the endosome membrane. The formation of the large endosomes is a result of fusion between smaller endocytic vesicles in a process that takes 1 min to complete (Figure 4(b)).

After 2 hours or longer, most anti-VEGFR2 was found in large endosomes, but colocalization with LTR was also frequently observed, indicating that the antibody had reached late endosomes/endolysosomes. The level of colocalization of anti-VEGFR2 with LTR, as evaluated by Pearson's correlation coefficient, increased with increasing times of chase with correlation coefficient values of 0.23±0.05 and 0.37±0.11 for 2 hours and 6 hours, respectively. Furthermore, after 120 min, the size distribution of anti-VEGFR2-containing compartments was altered: anti-VEGFR2 was present in large endosomes (merged image, green) as well as in smaller and more acidic vesicles in the perinuclear region (merged image, yellow), typical of late endosomes/lysosomes. Anti-VEGFR2 was seen in both membrane and in the lumen of these structures.

## 4. Discussion

In this study, we have shown the following: (1) Quiescent rat LSECs express VEGFR1 and VEGFR2 and endocytosis of VEGF-A is mainly mediated by these two receptors. (2) Treatments of LSECs with pharmacological agents that inhibit endosomal acidification (concanamycin A) and lysosomal proteases (leupeptin) indicated that endocytosed VEGF-A dissociates from its receptors in early endosomes and is subsequently routed to the lysosomes for degradation. (3) The essential role of clathrin-mediated endocytosis was seen by the effect of dynasore, a dynamin GTPase inhibitor. Dynamin-2 is essential for membrane fission and formation of clathrin-coated vesicles [37]. Its loss prevents VEGFR2 endocytosis, resulting in reduced angiogenesis [38]. Treatment of LSECs with a saturating concentration of VEGF-A showed that as much as 70-75% of the total cellular VEGFR2 may be susceptible to rapid VEGFA-mediated degradation, whereas VEGFR1 protein level was almost unaltered by the same treatment. (5) Finally, this study presents the internalization and intracellular trafficking of surface-bound fluorescently labeled anti-VEGFR2 Ab in LSECs.

Degradation of VEGFR2 is a consistent finding in VEGFA-stimulated endothelial cells. However, studies of endothelial cells have given different results regarding the extent and rate of VEGFR2 degradation upon exposure to saturating concentrations of VEGF-A (≥ 1 nM). For example, Duval et al. [36], similar to our results, found that VEGFR2 in BAECs undergoes almost complete degradation within 30 min of stimulation. Likewise, Gourlaouen et al. [34] found rapid (30-60 min) loss of VEGFR2 in HUVECs. Gampel et al. [39] also observed rapid (≤30 min?) degradation of VEGFR2 in HUVECs, but to a lesser extent (40%). Ewan et al. [28], using HUVECs, also reported an almost complete loss of VEGFR2, but only after 120 min of stimulation. Studies of the effects of VEGF-A on VEGFR1 degradation have, however, produced conflicting results. Some authors have reported no significant degradation of VEGFR1 in HUVECs [40, 41], whereas others have reported that VEGFR1 in HUVECs undergoes VEGF-A-stimulated degradation [42, 43]. It is not clear why the effects of VEGF-A on VEGF receptors vary so much across studies.

Immunoblot analysis of the cell lysates from LSECs treated with 1 nM unlabeled VEGF-A showed that as much as 70-75% of VEGFR2 is degraded within 30 min, whereas the VEGFR1 level was little changed after 120 min exposure to VEGF-A. These results suggest that following VEGF-A treatment these two receptors are sorted differently after entering into early endosomes. VEGFR1, like scavenger receptors, mannose receptors, and Fc*γ*RIIb2, is recycled, probably directly via tubular domains of the early endosomes, to the plasma membrane, whereas VEGFR2 is diverted into multivesicular bodies which fuse with terminal lysosomes and will be degraded in endolysosomes. Rapid downregulation of VEGFR2 in LSECs may reflect the fact that LSECs perform a more speedy endocytosis than most other cells that so far have been studied. It has been shown that ligands of scavenger receptors and mannose receptors in LSECs* in situ* or in suspension are internalized from the cell surface with a half time of about 20 sec [44].

The capacity of LSECs to endocytose VEGF-A at 37°C exceeded maximal 4°C binding several times for either of the receptors, implying that additional receptors are recruited from intracellular pools to the cell surface during the incubation at 37°C. The most likely explanation for this observation is that LSECs have a significant intracellular pool of VEGFR1 that can rapidly be mobilized to the cell surface in response to VEGF-A stimulation and then undergo repeated recycling. However, because 25-30% of VEGFR2 appears to be unaffected by degradation in response to VEGF-A, the possibility that this fraction of VEGFR2 may represent a pool of dynamically internalizing and recycling receptors that could contribute to the uptake of VEGF-A cannot be excluded. This conclusion is supported by the study conducted by Braet et al. [6], who have demonstrated through immunofluorescent studies that in rat LSECs VEGFR1 is predominantly intracellular with a perinuclear distribution. VEGFR2 and neurropilin-1 (NRP1), a coreceptor for VEGF-A, were also shown to have a perinuclear localization and faint intracellular staining.

The presence of intracellular pools of VEGFR1 and VEGFR2 may be a general phenomenon in vascular endothelial cells regardless of the level of receptor expression. For example, a study by Mittar et al. has shown that VEGFR1 in HUVECs is primarily an intracellular protein (the Golgi apparatus contains ~80% of VEGFR1) that rapidly traffics from the trans-Golgi to the plasma membrane. This rapid mobilization of VEGFR1 is dependent on VEGFR2-activation and mediated by calcium release from intracellular stores [41]. A study by Manickam et al. [45] has also shown that a fraction (~25%) of total VEGFR2 is localized to the Golgi compartment in quiescent HUVECs and that this pool of VEGFR2 is rapidly mobilized to the plasma membrane on VEGF-A_165_ stimulation. Another study has shown that approximately 40% of VEGFR2 in resting HUVECs constitutively recycles between the plasma membrane and early endosomal compartments (distribution similar to early endosomal marker EEA1) in a pathway that is independent of VEGF stimulation [46].

The biochemical results were corroborated and extended by studies that demonstrated that surface-bound fluorescently labelled anti-VEGFR2 Ab undergoes internalization and endosomal trafficking prior to its entrance in endolysosomes. However, intracellular trafficking of internalized anti-VEGFR2 in adherent LSECs was much slower than transport of internalized [^125^I]-VEGFA to lysosomes in cell suspensions. In the cultured cells hardly any VEGFR2 had reached endolysosomes after 30 min whereas 75% of the receptor was degraded after incubating suspended cells with VEGFA for 30 min. The slower transport of the receptor in adherent cultured cells than in cells* in situ* or cells in suspension is consistent with earlier studies showing that the endosomal trafficking of ligands for mannose and scavenger receptors to the lysosomal compartment in adherent LSECs is severalfold slower than in suspended LSECs or in LSECs* in situ*, although the general pattern of intracellular trafficking of ligands is similar between these systems [47].

A very prominent step in the itinerary of the VEGFR2 (as detected by fluorescently labeled anti-VEGFR2 Ab) is the early endosomes. They seem to be ring-shaped because the fluorescent Ab is attached to VEGFR2 in the endosomal membrane. The rapid increase in the size of early endosomes is part of a process that leads to the formation of late endosomes [48], and it is not restricted to LSECs that internalize VEGFR2. A similar process takes place in LSECs that endocytose surface-bound acetylated low density lipoprotein, which is mediated by scavenger receptors (unpublished data). Gradually more and more anti-VEGFR2 Ab was transferred to the lumen of the late endosome. The late endosome will fuse with a terminal lysosome and VEGFR2 will be degraded in an endolysosome.

## 5. Conclusion

To our knowledge, the current study provides the first quantitative analysis of receptor-mediated endocytosis of VEGF-A in quiescent LSECs. In addition, this study addresses an original question about the relative contribution of VEGFR1 and VEGFR2 to the endocytosis of VEGF-A in vascular endothelial cells and suggests VEGFR1 can play a significant role in mediating VEGF-A endocytosis in LSECs. However, our study has limitations, including that very low number of cell surface VEGFR1 in LSECs precluded monitoring the internalization and intracellular trafficking of fluorescently labeled anti-VEGFR1 Ab by confocal microscopy. In addition, this study only accounted for the uptake of VEGF-A via VEGFR1 and VEGFR2 and did not take into account other cell surface receptors that could potentially interact with VEGF-A at the cell surface and play a role in this process. However, it seems to be unlikely that other surface receptors, besides VEGFR1 and VEGFR2, can account for the two-fold greater uptake in control cells compared to cells that were incubated with VEGFR1-blocking peptide, because both the observations concerning significant reduction in [^125^I]-VEGF-A uptake in the presence of the VEGFR1-blocking peptide and those demonstrating rapid degradation of VEGFR2 could adequately explain our results and are more consistent with VEGFR1 recycling and reutilization. This is also consistent with the current idea that VEGFR1 is a decoy receptor serving as a mechanism for clearance of locally secreted (from hepatocytes) and/or locally released (from the extra cellular matrix) VEGF. VEGFR1 is well equipped to this function. It has extremely high affinity for VEGF-A and is internalized very rapidly.

## Data Availability

The biochemical and fluorescence microscopy data used to support the findings of this study are included within the article.

## Abbreviations

VEGF: Vascular endothelial growth factor
VEGFR: VEGF receptor
LSEC: Liver sinusoidal endothelial cell.
